# Impact of the wildland–urban interface on large carnivore damage in the Polish Carpathians

**DOI:** 10.1007/s13280-025-02201-0

**Published:** 2025-06-25

**Authors:** Dominik Kaim, Carlos Bautista, Michael Leitner, Franz Schug, Nuria Selva, Volker C. Radeloff

**Affiliations:** 1 Institute of Geography and Spatial Management, Faculty of Geography and Geology, Jagiellonian University, Gronostajowa 7, 30-387 Kraków, Poland; 2 Institute of Nature Conservation, Polish Academy of Sciences, Mickiewicza 33, 31-120 Kraków, Poland; 3 Department of Geography and Anthropology, Louisiana State University, Baton Rouge, LA 70803 USA; 4 SILVIS Lab, Department of Forest and Wildlife Ecology, University of Wisconsin-Madison, 1630 Linden Drive, Madison, WI 53706 USA; 5 Departamento de Ciencias Integradas, Facultad de Ciencias Experimentales, Centro de Estudios Avanzados en Física, Matemáticas y Computación, Universidad de Huelva, 21071 Huelva, Spain; 6 Estación Biológica de Doñana, Consejo Superior de Investigaciones Científicas, 41092 Seville, Spain

**Keywords:** Carnivores, Coadaptation, Human–wildlife interactions, The Carpathians, Wildland–urban interface, WUI

## Abstract

Large carnivore populations are recovering across Europe, likely influencing human–wildlife interactions in the areas where both human and carnivore activity overlap, like wildland–urban interface (WUI). We analyzed over 3500 cases of damage caused by brown bears, wolves, and lynx in the Polish Carpathians (2010–2017) to identify spatial and temporal hot spots and their determinants. Wolf damage was associated with sheep density and historical WUI, while bear damage correlated with permanent bear occurrence and historical WUI. Contemporary WUI showed a negative relationship with damage occurrences. Incident-level analysis revealed that forest cover was a key factor for both species, while wolf damage was linked to sheep density and bear damage to proximity to buildings (reflecting beehive locations). Our findings emphasize the need to integrate current landscape characteristics with land use legacies to develop proactive, site-specific policies that mitigate carnivore impacts and promote resilient coexistence.

**Supplementary Information:**

The online version contains supplementary material available at 10.1007/s13280-025-02201-0.

## Introduction

Despite the global decline of large carnivores (Ingeman et al. 2022), in some regions such as Europe, they are recolonizing parts of their historical ranges decades or even centuries after their prior extirpation (Gula et al. 2020; Cimatti et al. 2021; Bernardi et al. 2025). However, Europe’s landscapes are largely human-dominated, which poses challenges for the full recovery of large carnivore populations (Ripari et al. 2022). Furthermore, carnivores presence may be negatively perceived by people, for instance, due to livestock depredation or other interactions (Bautista et al. 2021; Davoli et al. 2022; Pop et al. 2023; Singer et al. 2023). Such human–wildlife conflicts may decrease human acceptance of large carnivores and can substantially affect policy recommendations and the conservation status of recovering species (Kuijper et al. 2019). Therefore, there is a need to better understand the wildlife and human adaptation strategies and to establish proper conditions for coexistence with these species (Carter and Linnell 2023).

Contacts between humans and carnivores are usually concentrated in areas where the activities of humans overlap with wildlife habitat. One type of such area is the wildland–urban interface (WUI), where natural vegetation is close to housing (Radeloff et al. 2005). Although the WUI has been associated mainly with wildfires (Bento-Gonçalves and Vieira 2020; Radeloff et al. 2023; Schug et al. 2023), it is the focal area of a wide range of human–environment relations including interactions between humans and carnivores (Bar-Massada et al. 2014; Jenerette et al. 2022). For instance, suburban and exurban WUI is where interactions between humans and wildlife are concentrated in northern New York State (Kretser et al. 2008). WUI areas in Colorado offer a risk–reward trade-off for cougars due to increase in prey availability, which is why cougars occasionally appear in WUI (Blecha et al. 2018). Similarly, cougars use residential WUI areas in Washington causing livestock depredation (Kertson et al. 2011). However, not all housing located close to natural areas is equally attractive for large carnivores. For instance, black bears recolonizing northwest Connecticut and appearing close to people preferred exurban areas over those characterized by either lower or higher housing density (Evans et al. 2017). Similarly, not all types of human–wildlife interactions occur in one WUI type. For instance, human health and safety concerns related to wolf–human contact are more common in WUI areas in Wisconsin, but this was not the case for livestock depredation (Olson et al. 2019).

The above-mentioned examples show that the WUI may be a focal area for human–wildlife interactions, representing various coadaptation strategies between people and wildlife, but most of the evidence is for US landscapes (Carter and Linnell 2016). That is unfortunate, because the recovery of large carnivores in Europe is occurring in very different landscapes. While the WUI is widespread in Europe (Bar-Massada et al. 2023), the history of European settlements is very different from that in the USA, and European WUI patterns are strongly affected by long-term historical legacies (Kaim et al. 2023). However, it is yet unknown how WUI corresponds to human–wildlife interactions and coexistence under European conditions, although research conducted in various parts of Europe suggests that both bears (Støen et al. 2015; Cimatti et al. 2021) and wolves (Theuerkauf et al. 2003; Cimatti et al. 2021) avoid settlement areas, or at least roads (Theuerkauf et al. 2007).

In the past, Central Europe, a region with higher carnivore densities than Western Europe (Chapron et al. 2014), was also subject to dynamic political and demographic changes that substantially affected land use (Munteanu et al. 2014; Affek et al. 2021; Pavlačka et al. 2023). Those land use changes created an opportunity for some wildlife species to occupy new areas (Fernández et al. 2012; Gula et al. 2020). For instance, the eastern part of the Polish Carpathians was depopulated after WWII, resulting in a WUI decline, and became one of the critical areas for carnivore persistence in Poland (Fernández et al. 2012; Niedziałkowski and Putkowska-Smoter 2020), and also a focal area for bison reintroduction (Ziółkowska et al. 2016).

Substantial population and land use changes in the past, resulting in various trajectories of landscape transformation over time, and the presence of different species of large carnivores make the Polish Carpathians an ideal area to examine the relationship between WUI and damage pattern caused by these species. On the one hand, there are areas that used to be WUI in the past, but are no longer settled, but on the other hand, many villages have grown, and traditional farming persists. In general though, nineteenth-century land use patterns with minimal forest represented the coadaptation archetype best explained by the eradication through the habitat degradation (zero sum losers) that is not consistent with coexistence between people and wildlife, but the abrupt population decline was as a tipping point that allowed wildlife to adapt to new environmental conditions (Carter and Linnell 2023). Just after the depopulation, the area represented the ‘fragile stability’ where human–wildlife interactions were negligible. Over time, ‘conservation reliance’, with gradual increase of population density over the next decades, accompanied by the subsequent land abandonment in various locations, and establishment of new protected areas, better explained the coadaptation approach (Carter and Linnell 2023).

For these reasons, our aim was to: (a) analyze how contemporary and historical WUI pattern affect the current spatial pattern of livestock and agricultural damage caused by large carnivores in the Polish Carpathians and (b) verify how WUI-related variables operate at various spatial scales.

## Materials and methods

### Study area

The Polish Carpathians are a mountain range reaching 2499 m. asl. with mosaics of forest and agriculture. Settlements are located mainly in valleys and more scattered than in other Carpathian countries (Kaim et al. 2022). The post-World War II forced resettlements in the eastern part of the Polish Carpathians in the 1940s, followed by a rapid forest cover increase from less than 40% to more than 70% of the study area, created a unique opportunity for large carnivores to recolonize that area (Fernández et al. 2012; Affek et al. 2021), being a tipping point for future human–wildlife coexistence. However, in the central and western part of the Polish Carpathians, carnivores were not extirpated in the past (e.g., in the Tatras), because the forested and inaccessible landscape offered them a relatively good habitat conditions (Nowak et al. 2008; Kutal et al. 2016; Hulva et al. 2018). Currently, habitat for wolves, lynx, and bears is concentrated in the southern, higher part of the Polish Carpathians rather than in the foothills, apart from the eastern part (Kaczensky et al. 2021).

Farming, including animal husbandry, in the area has declined over time, especially after the political and socio-economic transformation of the late 1980s (Munteanu et al. 2014; Bucała-Hrabia 2024). In the western part of the mountains, small-scale traditional farming used to dominate, which resulted in widespread land abandonment due to low profitability (Kolecka et al. 2017). In the eastern part, where the farmers were resettled in 1940s, the agriculture was converted to large, state-owned farms, which were privatized after 1990 (Lerman et al. 1999). Sheep husbandry is mainly maintained due to the cultural and biodiversity values of sustaining high nature value farming (Sendyka and Makovicky 2018), or the subsidies, and the cattle numbers are declining (Musiał and Musiał 2019). Large carnivore damage to livestock is compensated by the state, after formal reporting within 2 days since the damage occurred, and an officer, hunter, or veterinary doctor confirms that carnivore was responsible for the attack (Nowak et al. 2005; Gula 2008a, 2008b; Berezowska-Cnota et al. 2023). Prevention programs, such as electric fences or guarding dogs, are occasionally offered to farmers, typically as part of specific projects (Bautista et al. 2019).

### Damage data

**Fig. 1:**
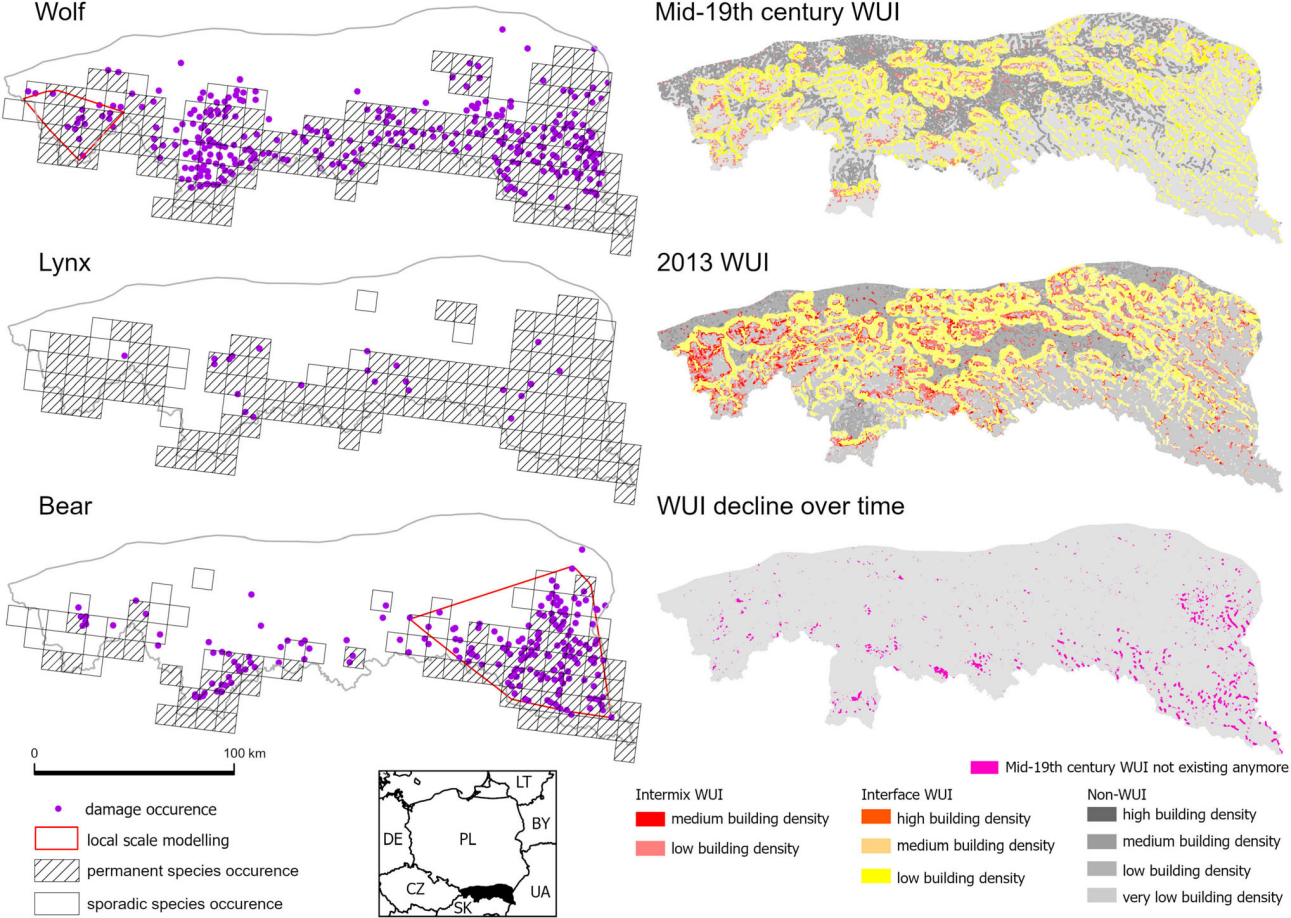
Damage, species, and WUI occurrence in the Polish Carpathians. Damage data show the proximate location at the village level, species occurrence based on Kaczensky et al. (2021). WUI data based on Kaim et al. (2024). *Note:* Nineteenth-century WUI map differs slightly due to political border change over time

We collected data on compensated damages caused by all large carnivore species (wolf, lynx and bear) from three Regional Directorates for Environmental Protection (*pol.* RDOŚ) for the provinces of Małopolska, Podkarpacie, and Śląsk for 2010–2017 (*n* = 3539 damage incidents in total). The spatial extent of the damage data covered the entire territory of the Polish Carpathians (ca. 20 000 km^2^; Fig. 1). The scope and form of the data differed slightly among the regions, because collection systems were not fully uniform and changed slightly over time (e.g., determining the location of the damage via GPS was not obligatory for most of the cases). For this reason, we geolocated the data to the village level so that the dataset was comparable across our study area. However, thematically the same data were collected regionally because each region in Poland compensates for the same type of damages. We also had to remove incidents if the location was missing or unclear, which slightly reduced the overall number of incidents (7% of the oldest wolf-related incidents). In most cases, the monetary value of the damage was listed and we retained that information (Table S2). We aggregated the damage from villages to the communes level (*n* = 194) and analyzed only those communes for which carnivore occurrence was reported from 2012 to 2016 (Kaczensky et al. 2021). This limited our study area to 140 communes for wolf damage and 100 communes for bear damage, and for lynx damage, a damage samples size was too small to allow for statistical analyses for that species (*n* = 38). Therefore, we still include lynx in the summaries (see Supplementary material), but detailed analyses were limited to wolves and bears.

Apart from the data aggregated at the commune level, we also obtained from RDOŚ the exact GPS locations for some of the wolf damage that occurred in the western part of the study area (2014–2019; *n* = 60, ca. 1800 km^2^), and GPS coordinates of the bear damage in Bieszczady, in the east (2014–2017, *n* = 293, ca. 4900 km^2^) from Bautista et al. (2021). We analyzed these datasets at the exact incident locations, independently from the data aggregated at the commune level.

### Spatial determinants of damage occurrence

We modeled where the damage occurred at two spatial scales. First, we explained the occurrence of damage at the commune level, which usually consists of several villages (mean area of the commune = 93 km^2^). This part of the analysis was performed based on the damage data collected for villages and aggregated to the commune level. For the subset of data with exact GPS locations of the damage occurrence, we analyzed the local context of the incidents using the exact locations as our units of analysis.

To analyze the spatial determinants that best explained the occurrence of damage we used the concept of the routine activity theory (RAT), which was developed for crime analysis. In that theory, an incident is most likely to occur when three independent aspects co-occur in time and space: an offender, a potential target, and the absence of a capable guardian or presence of attractive environment (Cohen and Felson 1979; Carter et al. 2017). We modified this theory slightly for the human–wildlife interactions framework in that we hypothesized that damage is most likely to occur if the area of activity of the potential ‘offender’ (carnivores), of the ‘target’ (damage subject), and an attractive environment co-occur in space and time. Our spatial determinants were selected to represent each of these three aspects (Table 1).

**Table 1:** Spatial determinants of the damage caused by large carnivores in the Polish Carpathians used at the commune level. ^*^2002 was the last year, where public statistics published the data on sheep number at the commune level; however, based on the county level from 2010 statistical data, we assumed that overall distribution and density of sheep in the area did not change substantially between 2002 and 2013

Variables	Explanation	Unit	Source
*Offender activity-related variables*			
Forest cover 2013	Forest cover share in commune 2013	%	Dobosz et al. (2019)
Persistent forest cover share 1860–2013	Share of forest that was persistent forest over the period 1860–2013	%	Kozak et al. (2018)
Forest cover dynamics 1860–2013	% forest cover in 2013, assuming 1860 forest cover was 100%	%	Kaim et al. (2016)
Resettlement of inhabitants after WW II	The communes depopulated in 1947 triggering rapid forest cover increase	Y/N	Ostafin and Kaim (2021)
Carnivore permanent occurrence	Permanent occurrence of bear or wolf in most of the commune area, based on 10 × 10 km square	Y/N	Kaczensky et al. (2021)
*Target activity-related variables*			
Sheep density in communes 2002*	Sheep density in the commune in 2002	Sheep/km^2^	stat.gov.pl
Cow density in communes 2020	Cow density in the commune in 2020	Cows/km^2^	stat.gov.pl
Average nearest neighbor among building 2013	Average distance to the nearest neighbor among buildings in the commune in 2013	m	BDOT 10k database
Building density 2013	Building density in commune	No. of houses/km^2^	BDOT 10k database
Building dynamics 1860–2013	% of buildings in 2013, assuming 1860 number of buildings was 100%	%	Kaim et al. (2021) BDOT 10k database
Tourist trail density	Density of tourist trails in the commune in 2013	km/km^2^	OpenStreetMap
Commuting rate	Number of people commuting to work in the commune per person leaving for work in 2011	Ratio	stat.gov.pl
*Attractive environment-related variables*			
Forest largest patch index 2013	How much area of the commune is occupied by the largest forest patch	%	Kaim et al. (2016)
Total WUI share 2013	WUI share in commune 2013	%	Kaim et al. (2024)
Past 1860 WUI	Share of WUI 1860, not WUI anymore in 2013	%	Kaim et al. (2024)
Forest in WUI share 2013	Forest located in WUI share in the commune 2013	%	Kozak et al. (2018; Kaim et al. (2024)
Medium-housing-density interface WUI share	Medium-housing-density interface WUI share in the commune in 2013	%	Kaim et al. (2024)
Low-housing-density interface WUI share	Low-housing-density interface WUI share in the commune in 2013	%	Kaim et al. (2024)
Low-housing-density intermix WUI share	Low-housing-density intermix WUI share in the commune in 2013	%	Kaim et al. (2024)
WUI 2013 already WUI in 1970 share	WUI 2013 already WUI in 1970 share in the commune in 2013 (short-term legacy effect)	%	Kaim et al. (2024)
WUI in 2013 already WUI in 1860 share	WUI 2013 already WUI in 1860 share in the commune in 2013 (long-term legacy effect)	%	Kaim et al. (2024)
WUI dynamics 1860–2013	% WUI in 2013, assuming 1860 WUI was 100%	%	Kaim et al. (2024)
Elevation range	Range of elevation within the commune—difference between the highest and the lowest point in the municipality (ALOS Global Digital Surface Model (Version 3.1)	m	Tadono et al. (2014)
Slope mean	Mean slope in the commune (ALOS Global Digital Surface Model (Version 3.1)	Degrees	Tadono et al. (2014)

We assumed that an attractive environment is best explained by WUI occurrence. The WUI is typically defined as an area where housing is close to the wildland vegetation and is divided into two types: intermix and interface. The intermix WUI is where buildings and wildland vegetation intermingle, and the interface WUI is where buildings are close to large wildland vegetation patches (Radeloff et al. 2005). Accordingly, we analyzed several WUI-related variables, including WUI maps representing the period close to the center of the damage data collection period (2013), past WUI occurrence (mid-nineteenth century), WUI persistence or decline over time, and WUI types based on housing density (Tables 1, 2; Kaim et al. 2024).

**Table 2:** Spatial determinants of the damage caused by wolves and bears in Maxent models at the exact damage occurrence level. ^1^In the wolf model correlation with forest cover share above 0.7, which resulted in removing elevation (forest cover is an important indicator of wolf habitat); ^2^in the bear model correlation with elevation above 0.7, which resulted in removing distance to the main roads (elevation is an important habitat predictor in mountain areas); ^3^correlation with sheep density above 0.7, which resulted in removing cow density (only ~ 7% of the wolf damage were cows)**;** ^4^2002 was the last year, where public statistics published the data on sheep number at the commune level, however, based on the county level of the 2010 statistical data, we assumed that overall distribution and density of sheep in the area did not change substantially between 2002 and 2013; ^5^in the bear model correlation with persistent WUI share above 0.7, which resulted in removing total WUI share (total WUI is less specific than other WUI-related variables)

Variables	Explanation	Species	Unit	Source
*Offender activity-related variables*				
Forest share 2013	Forest cover share in a 500 m buffer around the incident	Wolf, Bear	%	Dobosz et al. (2019)
New forests share (appearing between 1970 and 2013)	Share of forests cover, that appeared between 1970 and 2013, in a 500 m buffer around the incident	Wolf	%	Kozak et al. (2018)
Persistent forest cover share 1860–2013	Share of forests existing constantly since mid-nineteenth century within a 500m buffer around the incident	Bear	%	Kozak et al. (2018)
Elevation^1^	Elevation (ALOS Global Digital Surface Model (Version 3.1)	Wolf, Bear		Tadono et al. (2014)
*Target activity-related variables*				
Distance to 1860 buildings	Distance to buildings in 1860s	Wolf, Bear	m	Kaim et al. (2021)
Distance to 2013 buildings	Distance to buildings in 2013	Wolf, Bear	m	BDOT 10k database
Distance to the main roads^2^	Distance to the closest section of the main road in 2013	Wolf, Bear	m	BDOT 10k database
Distance to the tourist trails	Distance to tourist trail 2019	Wolf, Bear	m	OpenStreetMap
Cow density in commune 2020^3^	Cow density in the commune in 2020	Wolf	cows/km^2^	stat.gov.pl
Sheep density in commune 2002^4^	Sheep density in the commune in 2002	Wolf	sheep/km^2^	stat.gov.pl
*Attractive environment-related variables*				
Total WUI share 2013^5^	Share of WUI areas within a 500 m buffer around the incident	Wolf, bear	%	Kaim et al. (2024)
Past 1860 WUI	Share of WUI existing in mid-nineteenth century and non-existing later, within a 500m buffer around the incident	Wolf, bear	%	Kaim et al. (2024)
Medium-housing-density interface WUI share	Share of medium-housing-density interface WUI within A 500 m buffer around the incident	Wolf, bear	%	Kaim et al. (2024)
Low-housing-density interface WUI share	Share of low-housing-density interface WUI within A 500 m buffer around the incident	Wolf, Bear	%	Kaim et al. (2024)
Low-housing-density intermix WUI share	Share of low-housing-density intermix WUI within A 500 m buffer around the incident	Wolf, Bear	%	Kaim et al. (2024)
Persistent WUI share	Share of WUI existing constantly since mid-nineteenth century within a 500 m buffer around the incident	Bear	%	Kaim et al. (2024)

### Data analysis

All damage data were first divided by species and characterized quantitatively and qualitatively (grouped by the time—month and year of the incident and which type of domestic animal was attacked). Second, when possible, we analyzed the cost of compensation (for details, see Supplementary Material Table S2).

To analyze the spatiotemporal pattern of the damage between 2010 and 2017 at the commune level, we used emerging hot spot analysis, where the communes were used as aggregating units (Harris et al. 2017; ESRI ArcGIS Pro 2020). Emerging hot spot analysis is a space–time pattern mining technique that identifies spatial trends (by calculating Getis-Ord Gi*) and temporal trends (by applying the Mann–Kendall trend test**)**. We used the queen rule to determine spatial neighborhood and a 1-year time step to determine temporal neighborhood. The analysis reveals one of several potential spatiotemporal trajectories (e.g., new hot spot, intensifying hot spot, or persistent hot spot) and its level of statistical significance (*p* < 0.1; ESRI, Inc., Redlands, CA**).**

To explain the commune level occurrence of damage caused by wolves and bears, we used stepwise (exploratory) Ordinary Least Square (OLS) regressions based on our set of spatial determinants (Table 1), and damage density (i.e., number of compensated damage claims in each year divided by the area of the commune) as the dependent variable (Burnham and Anderson 2002). The method tested all possible models starting with one variable and adding another one, up to five variables in total, in which the variables were not collinear (VIF < 7.5). In total, we tested 55 454 models each for wolves and bears. Then, we checked how many times each variable was statistically significant in a model, and how often its effect was positive or negative. The analysis was conducted in ArcGIS Pro.

In order to assess the spatial determinants of the damage at the local level, for which we had the exact GPS location of the incident, we used Maxent (Phillips and Dudík 2008). The sets of variables used in this analysis step were similar to those employed in the previous OLS stepwise regression and on the RAT. However, because the GPS data were collected in relatively small areas, compared to the entire Carpathian territory, we recalculated the variables from commune level to the exact-location scale and limited the chosen variables to those which best characterize the local landscape conditions and land use history (Table 2). We started this step by checking whether the variables were collinear (*r* > 0.7) and, if so, excluded one based on expert decision (details: see Table 2). Second, we assessed the overall area under curve (AUC) of the model and defined the three most important variables based on the jackknife test for wolf and bear separately, taking into account the AUC values, if only this particular variable was taken into account in the model.

## Results

### Damage characteristics

The data collected by the Regional Directorates for Environmental Protection from 2010 to 2017 in the Polish Carpathians shown that lynx was only responsible for 1% of the incidents, whereas 85% were caused by wolves, and 14% by bears (Table S1). Wolves mainly attacked sheep (> 86% of the damage), while bears mainly attacked beehives (> 98% of the damage). Lynx mainly attacked sheep, but also occasionally European fallow deer (*Dama dama*) raised by farmers (Table S1).

The overall cost of the compensation provided by the regional administration (from Małopolskie and Podkarpackie provinces, more than 97% of the data in total) was mainly for damage caused by wolves, and only 25% for bear damage (Table S2). The cost of damage due to lynx attack was just 1% of the total. The overall compensation cost for 2010–2017 was 4,156,156 PLN, that is less than 1 million EUR (based on the April 2025 exchange rate).

The temporal pattern of damage within the year differed among the three species. Almost no damage was reported due to bear activities in January and February, when bears hibernate, and the winter period from December to April was when wolves damage was rare (Fig. S1). There was no clear pattern of damage due to lynx activity; however, in the spring and early summer, incidents were rare (Fig. S1).

### Spatial and temporal hot spot analysis

**Fig. 2:**
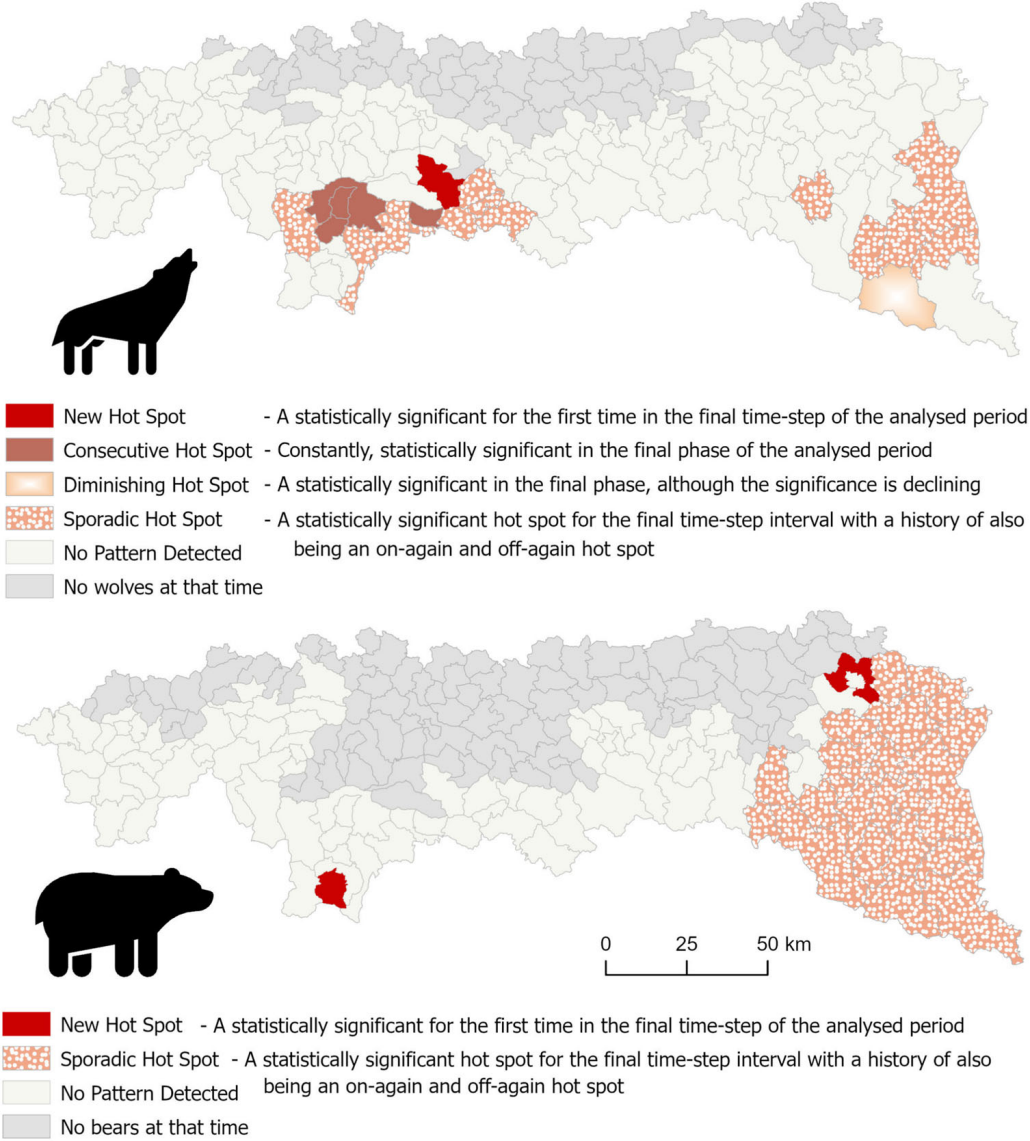
Emerging hot spots of damage in space and time caused by wolves and bears

Emerging hot spot analysis revealed that 121, out of the 140 communes where wolves’ were present, were not found as statistically significant hot spots (i.e., the damage levels in these communes are close to the average; 86% of the cases). The remaining communes formed two hot spots of damage caused by wolves in the central (e.g., Gorce Mountains and surroundings) and eastern parts of the Polish Carpathians, respectively. There were important temporal differences in damage pattern between these two areas, however. According to the emerging hot spot method terminology, in the central area, most of the communes were either sporadic hot spots (i.e., a commune was a hot spot in the final year and also at least on prior year) or consecutive hot spots (i.e., a hot spot in at least the last two years). The central part of the mountains was also, where there was also the only new hot spot (never being a hot spot in the previous years) commune in the dataset. In the eastern area, most of the communes were sporadic hot spots, except for a diminishing hot spot observed (the statistical significance of the hot spot in the commune decreased over time; Fig. 2).

For bear damage, there were no hot spots in 76 out of the 100 communes where bears were present (i.e., no spatiotemporal dependency was detected in 76% of the communes). The remaining communes were mainly sporadic hot spots and located almost exclusively in the eastern part of the Polish Carpathians. Two communes were classified as new hot spots (i.e., they were only statistically significant in the last year), one in the northeast, and the other in the Tatra Mountains (Fig. 2).

### Spatial determinants of damage occurrence—commune level

Our regression models showed that sheep density was the most important variable explaining wolf damage (statistically significant in all the models that included it, and its effect was always positive on damage density), while WUI-related variables were the second and third most important variables (Table 3). Specifically, these variables were the WUI areas that disappeared since 1860 (Past 1860 WUI), and the 2013 WUI shares, with the first being significant in more than 96% of the models and all had a positive effect, and the latter being significant in more than 64% of the models and of those in 97% of the cases with a negative effect (Table 3).

**Table 3:** Variable significance (the percentage of our regression models in which a given variable was a candidate variable and significant; total *n* = 55 545) and the direction of its effect in regression models (only the three most important variables for each species are shown)

Variable	% significant	% negative	% positive
*WOLF*			
Sheep density	100	0	100
Past 1860 WUI	96.41	0	100
WUI in the commune in 2013	64.34	97.66	2.34
*BEAR*			
Permanent bear occurrence	100	0	100
Past 1860 WUI	89.29	0	100
Forest in commune in 2013	59.20	0.28	99.72

Our models for bear damage showed that the most important variable explaining current damage density at the commune level was permanent bear occurrence (statistically significant in all the models, always with a positive effect), while the second and third most important variables were statistically significant in nearly 90%, and 60% of the models they appeared in, respectively (Table 3). 1860 WUI not existing currently had always a positive effect, and similarly forest share in commune in 2013 was almost always positively correlated with damage incident density (Table 3).

### Spatial determinants of damage occurrence—exact incident location

**Fig. 3:**
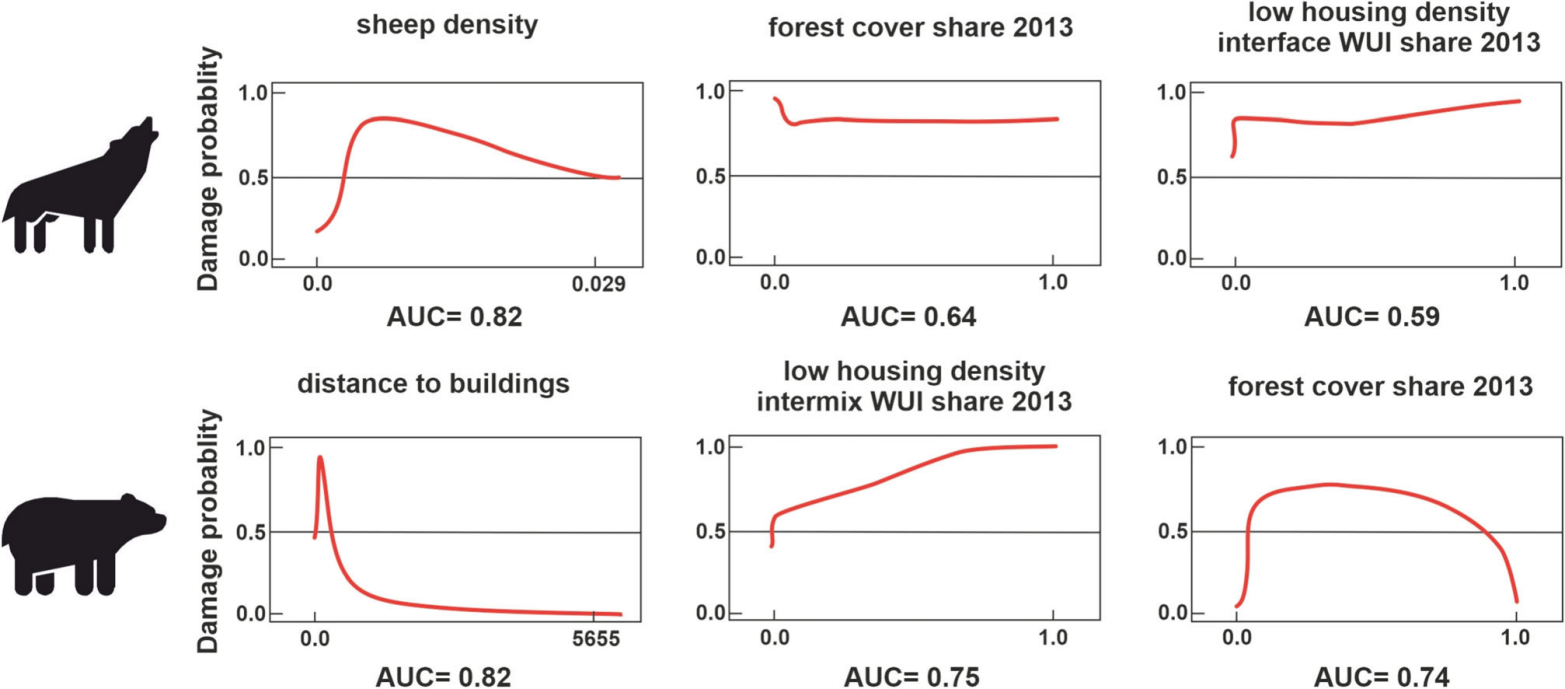
The three most important variables performing best on the AUC values, if only those variables are included in the model, explaining wolf and bear damage probabilities at the exact incident location based on Maxent models

The Maxent models for the exact damage locations by wolves performed well (AUC = 0.877) and were consistent with the regression-based results at the commune level. For the location of wolf damage, sheep density was again the most important variable, while contemporary percent forest around the incident and contemporary low-housing-density interface WUI share were slightly less important variables (Fig. 3). Bear damage models performed equally well (AUC = 0.891), and the most important variables were current distance to the nearest building, current share of low-housing-density intermix WUI, and forest cover (Fig. 3).

## Discussion

We analyzed large carnivore damage in the Polish Carpathians, demonstrating the applicability of routine activity theory. First, wolf incidents occurred in high-sheep-density areas, while bear incidents were linked to beehives near buildings in low-housing-density intermix WUI (Fig. 3), both indicating the area of ‘target activity’. Second, damage locations were within an attractive environment, characterized by high forest cover and historical, rather than current, WUI. Former WUI areas, now depopulated villages or pastures, remain wolf damage hotspots. Currently, these areas are still, at least partly, used for sheep grazing, although the density of shepherd’s huts is now much lower now (Tokarczyk 2018; Sobala 2023), resulting in a decline of WUI. High shares of pastures and forests increased the probability of wolf damage also in other studies conducted in the Polish Carpathians (Fedyń et al. 2022). For bears, permanent occurrence aligned with 1860 WUI and low-housing-density intermix WUI, indicating forest-dominated areas. The only contemporary WUI variable positively correlated with wolf damage was low-housing-density interface WUI, where sheep grazing occurs near villages and forests. This makes the Carpathians different from the US landscapes where black bear conflicts are common, and where medium housing density is where damages are concentrated (Baruch-Mordo et al. 2008; Klees van Bommel et al. 2020). The reason for this difference is probably that the type of conflict differs: while beehives damage dominates in the Polish Carpathians, in the USA, black bears mainly cause damage when searching for garbage or other human food resources (Evans et al. 2017; Klees van Bommel et al. 2022). Both wolves and bears in Europe usually avoid humans (Theuerkauf et al. 2003; Bautista et al. 2021; Cimatti et al. 2021), and the importance of low-housing-density WUI as a positive predictor in our analysis confirm this interpretation (Fig. 3).

Our models results showed that WUI is an important predictor of negative interactions such as livestock predation or attacks on beehives. However, which WUI-related variable mattered most, varied among species which caused losses, and the damage they caused. Our results at the commune level indicated for both wolf and bear damage, historical WUI not existing anymore was an important determinant, positively correlated to damage occurrence, while contemporary WUI was negatively related and less important. The exact incident locations also highlighted the role of damage type (e.g., sheep attack, beehive damage), and that of more nuanced WUI subtypes in damage occurrence explanation. Forest cover share was also among the crucial spatial determinants for both species (Fig. 3). This shows that while damage type is a connected to the landscape context of the incident (sheep attack on the pasture located out of the village, and destruction of beehive located in the garden close to the forest), the incident could only happen if the overall habitat enabled the carnivores presence. That is why past WUI and permanent bear occurrence tend to be more correlated to the damage, as they show the areas where human presence declined over time, creating the opportunity for carnivores to recover.

Contemporary WUI share, in general, was negatively correlated to the damage, showing that the carnivores tended to avoid people, which coincides with previous studies in the region (Theuerkauf et al. 2007; Bautista et al. 2021), other parts of Europe (Cimatti et al. 2021) or the US (Barker et al. 2023). The above-mentioned results suggest that the contemporary depopulated and abandoned areas of Europe, with declining WUI share, create an opportunity for future habitats for carnivores (Lasanta et al. 2017). However, those areas may also be likely conflict locations. It shows the importance of including long-term WUI trajectories (Kaim et al. 2024) as spatial determinants of contemporary damage occurrence, which had not been done before. Although contemporary WUI data are available globally (Schug et al. 2023), the historical WUI maps are missing. However growing amount of historical land use data, and past building footprints (Uhl and Leyk 2022; Szubert et al. 2024) will make it possible to create such maps in future. It would be especially useful in the areas, where widespread depopulation and large carnivore recovery took place.

We found that although a substantial part of the Polish Carpathians was within the wolf area of activity, there were only two clear damage hot spots, in the eastern and central parts of the mountains, respectively, indicating a large area of human–wildlife coexistence. Overall, hot spots appear intermittently over the years. However, in the central part of the mountains, hot spots have intensified over time, while in the east, they have started to fade. We speculated that this was the case, because sheep numbers increased slightly in both central and western areas from 2010 to 2020, but decreased slightly in the eastern part of the mountains.^1^ While an increase in hot spot significance at the commune level can be linked to many potential factors (e.g., an increase in wolf density, increase in sheep density or increase in farmer awareness of compensation schemes), a decrease in significance is more difficult to explain, but might be also due to either an increase in the wild prey population or better sheep protection, and farm adaptation (Gula 2008a).

The main bear damage hot spot was located in the eastern part of the Polish Carpathians, and was very consistent over time. Additionally, two small new hot spots occurred in the Tatra Mountains and in the foothills of the eastern Carpathians, the northeastern limit of our study area, respectively. Although the Tatra bear population lives mainly in the Tatra National Park, the area is under very high human pressure for various reasons (e.g., high tourist pressure and urban development close to the national park border), and bears sometimes visit nearby settlements, resulting in interactions with humans (García-Rodríguez et al. 2021). In the northeastern foothills of the Carpathians, bear presence is rare, because this is the periphery of their range (Fernández et al. 2012; Chapron et al. 2014), and there were no damage incidents until recently (Berezowska-Cnota et al. 2023). This may suggest that bears started to use more consistently an area that lacks a tradition of coexistence with bears and thus, where beehives are not protected, suggesting the need to focus on adaptation measures in the future (Carter and Linnell 2023). However, overall, only permanent bear occurrence areas were associated with bear damage. Still, other areas of bear occurrence during recent decades have not experienced any statistically significant increase in reported damage indicating the existence of some coadaptation strategies. This may indicate that there was no increase in the occurrences of bear damage during the study period. An alternative explanation of such pattern is that a potential increase of bear damage due to an increasing bear population or food conditioning by bears was buffered by increasing efforts to prevent damage. However, that does not fit with the reality of our study system, where subsidizing preventive measures is still not the management priority, and the focus is on damage compensation (Bautista et al. 2021). That business-as-usual approach in damage management could explain the status quo in the occurrence of conflict hot spots and why they did not decrease during the study period and that yet new hot spots emerged.

The damage data showed that wolves accounted for the majority of the reports (nearly 85%), while bears were responsible for a substantially lower number of incidents (14%), and lynx was responsible for only 1% of the reported incidents. The number of damage reports per individual in the respective population, however, did not differ greatly among species. In the Polish Carpathians, wolves mainly attacked sheep (86%), while cattle and other species were reported in only 14% of all incidents. This value was similar in the past in the eastern Carpathians (Gula 2008b) and is similar to Poland in its entirety, although in the eastern lowlands, cattle are attacked more often (61% of the attacks) than sheep (24%) (Fedyń et al. 2022). At the European level, cattle and goats are more affected (19.5% and 11%, respectively), and sheep are less affected (54.2%), than what we found (Singer et al. 2023). This might be partly because sheep are more common in mountains, such as the Carpathians. Indeed, in Italy’s northern Apennines sheep were also attacked more often (69.4%) than on average in Europe (Davoli et al. 2022; Singer et al. 2023). Effective sheep protection strategies and, more broadly, effective policy toward sheep in mountain areas are crucial for limiting human–wolf conflicts (Bruns et al. 2020; Kutal et al. 2023).

The reported bear-caused incidents were almost all related to beehive damage. This makes the Carpathian case different from bear-related damage in the Apennines, Slovenian Alps, and Greek Pindos or in Norway, where livestock damage is more common (Bautista et al. 2017), while damage to crop or garbage bins are more common in other continents (Can et al. 2014). Lynx-related damage was relatively rare, but sheep were again the most targeted livestock species. Nevertheless, the presence of European fallow deer in lynx-caused compensation reports shows that introducing farmed deer to the mountainous landscape, where carnivores have been present for decades, entails a high risk.

The frequency of the incidents over the course of the year differed substantially among the carnivore species. Wolves caused damage especially from May to November, which is when sheep graze in the mountains. The peak of attacks occurred from August to October, which is typical for northern Europe, too (Singer et al. 2023). Bear damage was concentrated from April to November, but in the most recent years also in December and March. The temporal variation in the occurrence and intensity of damage depends on several conditions, such as the availability of natural resources or artificial feeding (Sergiel et al. 2020; Bautista et al. 2023), and we caution against linking the extended damage period in winter directly to climate change. However, we cannot rule this out either and the frequency of bear winter observations in the region has increased (Bojarska et al. 2019). The low number of incidents of lynx damage made it difficult to assess temporal patterns, although from February to July, incidents were less common than in the latter part of the year. In other parts of Central Europe, lynx predation on domestic species was also a minor issue (Belotti et al. 2015).

Past and future land use trajectories shape human–wildlife coadaptation archetypes (Carter and Linnell 2023). In the eastern Carpathians, high pre-WWII human density and low forest cover led to archetypes misaligned with coexistence (‘reciprocal damages’ or ‘eradication’). Post-WWII resettlements triggered population declines and rapid forest expansion, creating favorable conditions for wildlife recovery. Combined with conservation efforts and species protection, this shift fostered coexistence-compatible archetypes. Between 2010 and 2017, most Carpathian communes saw slight population growth, except in the eastern region, where carnivores are most abundant and population declined. Looking ahead to 2050, further declines are projected in the east (Price et al. 2017), while elsewhere, settlement and forest cover expansion will increase WUI. Many new settlements will be tourism-driven, potentially fostering a ‘sustained co-benefits’ archetype. However, increased carnivore encounters may trigger negative perceptions, leading to maladaptive archetypes. A ‘conservation reliance’ archetype is also possible but would require effective preventive measures and a robust compensation system for farmers (Bautista et al. 2021; Carter and Linnell 2023). Furthermore, human attitudes and coadaptation strategies may vary considerably among species, and the presence of wolves may be perceived differently by society than that of bears (Sevillano-Triguero et al. 2023), potentially requiring divergent conservation strategies. The overall perception of wolves is more negative than of bears, and recent political moves have sought to lower the level of wolf protection in Europe (Ordiz et al. 2024). This underscores the role of governance in shaping land dynamics and coexistence resilience, potentially shifting the system toward reciprocal damage.

## Conclusion

We found that even though carnivores live throughout a substantial part of the Polish Carpathians, damage hot spots are fairly concentrated. The probability of wolf damage is related mainly to the density of sheep, and damage by bears to the locations of beehives, and for both species in areas that are highly forested. We also found that past WUI not existing today exerts important legacies that explained damage occurrence, which had not been considered in prior studies. Our research contribute to the ongoing discussion on the importance of contemporary depopulating areas of Europe as future animal habitats, where the shift in human–wildlife coexistence archetype may take place. The coadaptation archetypes are not static in space and over time, and can evolve differently, depending also on the history of the coexistence or its lack, in the areas of the potential expansion.

## Supplementary Information

Below is the link to the electronic supplementary material.

Supplementary file1 (PDF 358 KB)

## Data Availability

Input data for the commune level analysis, and exact GPS location of wolf damage are available at 10.5281/zenodo.15439390. Bear damage exact locations are available at https://doi.org/10.5061/dryad.rfj6q57bc. WUI raster maps are available at https://doi.org/10.5281/zenodo.10135054.

## Footnotes

1 https://bdl.stat.gov.pl/.
