# Supplementary material for: Impact of the wildland–urban interface on large carnivore damage in the Polish Carpathians
Source: Ambio. 2025 Jun 25;55(5):1011–25. doi: 10.1007/s13280-025-02201-0 (PMC13035969; doi:10.1007/s13280-025-02201-0)
Supplement: Supplementary file 1 — Supplementary file1 (PDF 358 KB) [file 13280_2025_2201_MOESM1_ESM.pdf]

AMBIO

Supplementary Information: This Supplementary Information has not been peer reviewed

Title: Impact of the wildland-urban interface on large carnivore damage in the Polish Carpathians

Authors: Dominik Kaim, Carlos Bautista, Michael Leitner, Franz Schug, Nuria Selva, Volker C. Radeloff

Tab. S1. Damage (no. of attacks) caused by wolves, lynx, and bears in the Polish Carpathians 2010-2017 that the Regional Directorates for Environmental Protection paid compensation for.

	**target**	**No.**	**[%]**
**bear**	sheep	8	1.56
	cattle	0	0.00
	beehives	505	98.25
	other/unknown	1	0.19
	SUM	*514*	*100*
**lynx**	sheep	22	57.89
	cattle	1	2.63
	other/unknown	15	39.47
	SUM	*38*	*100*
**wolf**	sheep	2623	86.20
	cattle	235	7.72
	other including:	185	6.08
	dog	42	1.38
	horse	53	1.74
	goat	49	1.61
	other/unknown	41	1.35
	SUM	*3043*	*100*
**Total sum**		**3595**	

Tab. S2 The total cost of the damage based on the data from małopolskie and podkarpackie voivodeships (data for śląskie was not available, although they account for less then 3% of the incidents; the value of incidents with total available damage cost is shown in brackets next to the species name). Conversion to EUR based on the April 2025 exchange rate.

	PLN	EUR*	[%]
**bear (98%)**	1,080,450	250,280	26
**lynx (97%)**	46,871	10,857	1
**wolf (98%)**	3,028,836	701,614	73
**SUM TOTAL 2010-2017**	4,156,156	962,751	

*The value was averaged, based on the assumption that 1 EUR = 4.30 PLN

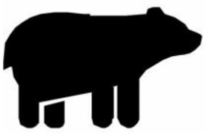

	Jan	Feb	Mar	Apr	May	Jun	Jul	Aug	Sep	Oct	Nov	Dec
**2010**	0	0	2	21	2	6	30	18	13	2	2	4
**2011**	2	0	2	6	11	13	7	4	4	7	3	4
**2012**	3	0	4	20	1	7	2	4	1	4	5	2
**2013**	0	0	3	12	6	4	3	5	1	0	1	1
**2014**	0	0	4	7	7	10	8	5	12	7	1	0
**2015**	0	0	0	3	6	2	2	1	6	3	5	3
**2016**	1	2	4	4	4	7	5	4	5	4	2	6
**2017**	3	2	10	4	16	9	11	21	11	15	16	8

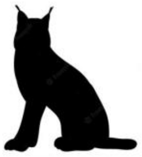

	Jan	Feb	Mar	Apr	May	Jun	Jul	Aug	Sep	Oct	Nov	Dec
**2010**	0	0	0	0	0	0	0	0	0	1	1	0
**2011**	0	0	0	0	0	0	1	0	1	0	1	0
**2012**	0	0	0	1	1	0	0	0	0	1	0	0
**2013**	0	0	0	0	1	1	0	0	0	0	0	1
**2014**	0	0	1	0	1	0	0	0	0	0	1	0
**2015**	0	0	1	0	0	0	0	0	1	1	1	2
**2016**	2	0	0	0	0	0	0	1	1	1	1	0
**2017**	2	0	1	0	0	0	1	2	2	4	0	0

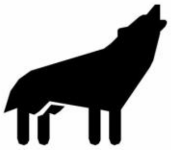

	Jan	Feb	Mar	Apr	May	Jun	Jul	Aug	Sep	Oct	Nov	Dec
**2010**	2	0	1	5	22	28	35	53	48	30	28	1
**2011**	4	1	1	9	31	30	50	56	63	53	27	9
**2012**	2	1	1	12	39	33	65	78	73	76	22	3
**2013**	3	1	0	3	38	32	45	49	75	50	24	7
**2014**	2	2	1	14	33	57	47	84	112	77	47	10
**2015**	0	1	1	13	34	42	51	80	84	71	39	9
**2016**	4	4	1	11	43	25	50	54	85	72	32	1
**2017**	2	1	5	9	28	38	71	68	90	91	26	4

Fig. S1. Temporal distribution of the damage (no. of attacks) of the three species reported from 2010 to 2017.
